# Dosimetric comparison study of ultrahypofractionated photon versus proton treatment plans in post breast-conserving surgery breast cancer

**DOI:** 10.1371/journal.pone.0344699

**Published:** 2026-03-11

**Authors:** Kitwadee Saksornchai, Tanawat Tawonwong, Puntiwa Oonsiri, Mananchaya Vimolnoch, Thanaporn Sarsitthithum, Chawalit Lertbutsayanukul, Chonnipa Nantavithya

**Affiliations:** 1 Division of Radiation Oncology, Department of Radiology, Faculty of Medicine, Chulalongkorn University, Bangkok, Thailand; 2 Division of Radiation Oncology, Department of Radiology, King Chulalongkorn Memorial Hospital, Thai Red Cross Society, Bangkok, Thailand; Northwestern University Feinberg School of Medicine, UNITED STATES OF AMERICA

## Abstract

**Background:**

There is an increasing utilization of ultrahypofractionation radiotherapy plans in whole breast radiotherapy. This study presents a comparative analysis of ultrahypofractionation using volumetric modulated arc therapy (VMAT) and intensity modulated proton therapy (IMPT) in treatment planning for patients undergoing breast conserving surgery (BCS).

**Materials and methods:**

CT datasets of twenty patients undergoing BCS, with ten on the left side and ten on the right side, were retrospectively replanned using both VMAT and IMPT techniques. The study included four scenarios: left/right breast only and left/right breast with regional nodes. A total dose of 26 Gy(RBE) was prescribed in 5 fractions. The CTV (clinical target volume) was optimized for IMPT, incorporating a 3 mm setup uncertainty and a 3.5% range uncertainty. The planning target volume (PTV) was used for VMAT optimization and evaluation for both techniques. Dose to the target volume and organs at risk (OARs) between IMPT and VMAT was analyzed.

**Results:**

The PTV-D95, along with the V10 and V20 of the ipsilateral lungs, was comparable between VMAT and IMPT plans. The ipsilateral lung Dmean and V5 were significantly lower for the IMPT methods for breast-only radiotherapy and for the right breast with regional nodes. IMPT plans showed significantly reduced Dmean, V5, and V10 doses to the contralateral lung, as well as Dmean and V5 doses to the heart in all subgroups. The Dmean of the heart was less than 1 Gy for the left-sided and less than 0.5 Gy for the right-sided subgroup in IMPT plans. However, the IMPT plans showed a significantly higher Dmax for the skin surface in all subgroups, as well as for the esophagus in the left breast with regional nodes subgroup.

**Conclusion:**

IMPT plans significantly reduced radiation exposure to most surrounding OARs compared to VMAT. Clinical outcomes are needed to confirm the potential for reduced late toxicities. However, this benefit was accompanied by an increased skin surface dose and, in specific cases, a higher esophageal dose.

## Introduction

Breast-conserving surgery (BCS) followed by radiotherapy has become the standard treatment for breast cancer [1–3]. The locoregional control and survival outcomes of breast-conserving surgery followed by radiotherapy are comparable with mastectomy while BCT could achieve more favorable cosmetic outcomes. To reduce overall treatment time of radiotherapy course, hypofractionated radiotherapy, e.g., 15–16 fractions, has been accepted as a standard fractionation regimen with the comparable oncological and cosmetic outcomes to the 5-week conventional fractionation. [4–7]. Furthermore, the recent published study of ultra-hypofractionation in early-stage breast cancer showed that 5 fractions over 1 week regimen was non-inferior to the moderate hypofractionation, 15 fractions over 3 weeks [8]. With shorter radiotherapy course, the ultra-hypofractionation offers more convenience to patients and less workload to health care workers, especially in the COVID-19 era. However, the published data on ultrahypofractionated radiotherapy did not include regional nodes in the target volume. Therefore, the side effects from regional nodes radiotherapy with ultrahypofractionation are still questionable. Reducing dose to adjacent organs is essential to ensure acceptable toxicities.

Several techniques have been proposed to reduce dose to organs at risk. While inverse planning photon techniques, e.g., intensity modulated radiotherapy (IMRT) and volumetric arc therapy (VMAT), could reduce high dose to organs at risk, these techniques may increase low dose to organs at risk compared with three-dimensional conformal radiotherapy (3D-CRT) [9,10]. Proton beam therapy, due to its Bragg peak, has been shown in several dosimetric studies to deliver lower doses to organs at risk compared with photon plans [11,12]. Clinical studies of proton beam therapy in conventional fraction also showed acceptable high-grade toxicities and were feasible for treating breast cancer [11,13,14]. To reduce dose to adjacent organs in ultrahypofractionated radiotherapy, the use of proton beam should be safe and acceptable. The recent dosimetric study of ultrahypofractionated regimen from our institution showed that proton significantly reduced the dose to organs at risk compared with photon in postmastectomy treatment planning, except higher dose to skin and esophagus [15].

Typically, patients who choose breast-conserving surgery usually require good cosmetic outcomes. Accordingly, dose to the skin is an important factor to predict possible side effects. However, the skin toxicity is usually mild and might not affect the quality of life or health condition. In contrast, radiotherapy-related toxicities of other adjacent organs to breast, e.g., heart, lungs, could cause serious health issues. Choosing a radiotherapy technique that minimizes side effects would provide the most benefit.

Although our previous dosimetric study demonstrated the advantage of proton beam in reducing doses to several OARs, the relatively higher skin dose remains a concern, particularly for intact breast treatments. Moreover, the target volumes of intact and postmastectomy breasts differ considerably, especially in thickness, making it difficult to directly apply the findings from postmastectomy cases to intact breast scenarios. Therefore, to better understand the dose distribution to the target volume and adjacent organs in the ultrahypofractionated regimen for intact breasts, we conducted this dosimetric study to compare proton therapy with photon therapy in breast-conserving surgery patients.

## Materials and methods

The retrospective dosimetric study involved CT datasets of twenty post breast-conserving surgery patients who had previously undergone breast radiotherapy at King Chulalongkorn Memorial Hospital in 2018–2019. Ten patients had right-sided breast cancer, while the remaining ten had left-sided breast cancer. The patients’ CT datasets were re-planned using both VMAT and IMPT approaches. The CT scans were acquired using a Siemens SOMATOM Definition AS 64-slice scanner from Siemens in Erlangen, Germany. Patients were positioned supine with a face straightand arms above the head, and immobilized with Vac-Lok (CIVCO Medical Solution, Iowa, USA) during data acquisition. A dose of 26 Gy (RBE) was prescribed in 5 fractions, with a generic relative biological effectiveness (RBE) value of 1.1 used for proton plans.

For each case, two sets of clinical target volumes (CTV) were generated, one including the breast only, and the other including the breast with regional lymph nodes, comprising axillary levels I, II, and III, supraclavicular lymph nodes, and internal mammary nodes (IMNs). The CTVs were re-contoured and reviewed by a group of specialized radiation oncologists who were investigators in this project for consensus following the Radiotherapy Comparative Effectiveness (RADCOMP) atlas [16]. The organs at risk evaluated were the ipsilateral and contralateral lungs, contralateral breast, heart, esophagus, thyroid, and skin surface. The radiotherapy technique has been described in detail previously [15]. Briefly, a 5-mm expansion from the CTV was added to the PTV but 5 mm was subtracted from the skin surface and 3 mm from the lung/chest wall interface. A 5 mm layer of skin was generated beneath the body’s surface. The Eclipse treatment planning system version 15.6 (Varian Medical System, Inc., Palo Alto, CA) created all the treatment plans. Four scenarios were created: left breast, left breast with regional nodes, right breast, and right breast with regional nodes.

All treatment plans were re-planned. In total, 40 VMAT plans were created, 10 for left breast only, 10 for left breast with regional nodal irradiation, 10 for right breast only, and 10 for right breast with regional nodal irradiation. Similarly, a total of 40 IMPT plans were generated, with 10 plans for each of the corresponding treatment categories. The VMAT plans were optimized with 6 MV photon beams. We used 4 arcs with the gantry running from about 240° to about 50° and from about 135° to about 310° for the right and left breasts, respectively. The collimator was set at an angle of 90° for 2 arcs, splitting the jaw to encompass the upper and lower part of the PTV. For each IMPT plan, two en-face fields of an anteroposterior (AP) field (0°) and an anterior oblique field with a gantry angle ranging from 30° to 45° were used. A 5 cm range shifter was used to modify the beam dosimetric coverage at all depths. The position of the proton treatment head (snout) was fixed at 42.1 cm for all plans. The plan was robustly optimized on CTV volumes with 5‐mm setup uncertainty and 3.5% range uncertainty. The proximal margin to the CTV was set to 0.1 cm, with spot spacing in the scanning direction and spacing between scanning lines set to 0.5 cm. The plans were normalized to 95% of the PTV being covered by 95% of the prescription dose.

Dose constraints for both VMAT and IMPT optimization are shown in S1 Table. Regarding the differences in physical characteristics between protons and photons, if the dose to OARs cannot be achieved, especially in VMAT group, the priority for optimization in both groups would be the target volume coverage. All plans were reviewed by radiation oncologists and medical physicists who were investigators in this project.

We reported the mean dose (Dmean), volume receiving 5 Gy (V5), volume receiving 10 Gy (V10), and volume receiving 20 Gy (V20) for the heart, ipsilateral lung, and contralateral lung. The maximum and mean doses for the thyroid, esophagus, and skin were documented. Version 1.5.1 of STATA (StataCorp LLL, Texas, United States) was used for statistical analysis. The paired t-test was utilized when the data followed a normal distribution. The Wilcoxon signed-rank test was used to compare the dose-volume histogram (DVH) between VMAT and IMPT approaches in scenarios where the data deviated from a normal distribution. A P value less than 0.05 was deemed statistically significant.

The study was conducted in accordance with the Declaration of Helsinki (as revised in 2013), and all patients’ data were anonymous. Since this manuscript is the dosimetric part of this protocol which we conducted based on the CT datasets, there was no intervention nor direct contact with the patients. Therefore, no informed consent was obtained for this part of the project. The study protocol was approved by the Research Ethics Committee of King Chulalongkorn Memorial Hospital (IRB approval number: 017/64) and data for dosimetric study part were accessed after approval (during May 15, 2021 – September 30, 2021).

## Results

**Fig 1:**
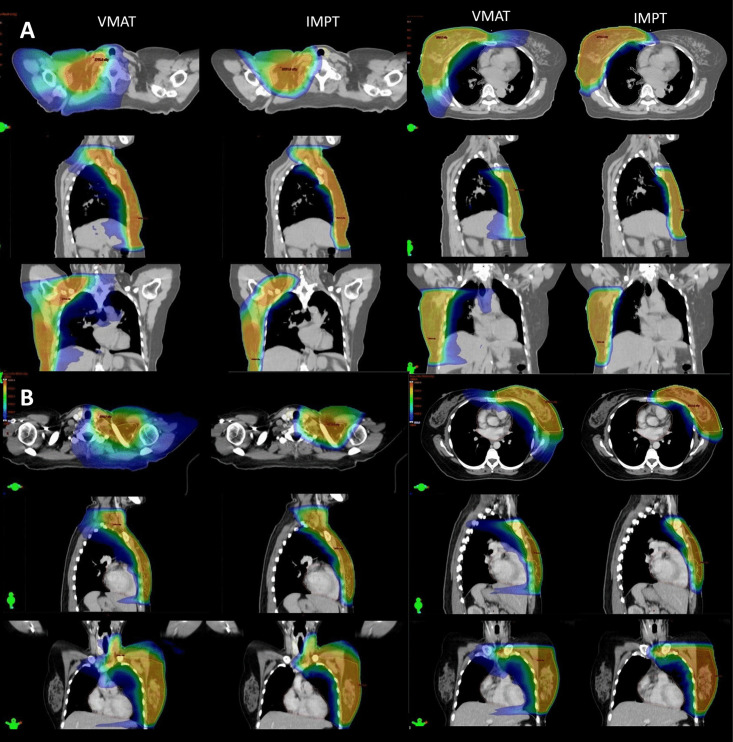
Dose distribution for the breast with and without regional nodes for volumetric modulated arc therapy (right side) versus intensity modulated proton therapy (left side). A Right breast and regional nodes. B Left breast and reginal lymph nodes. The axial, sagittal, and coronal images are presented from top to bottom, respectively.

**Fig 2:**
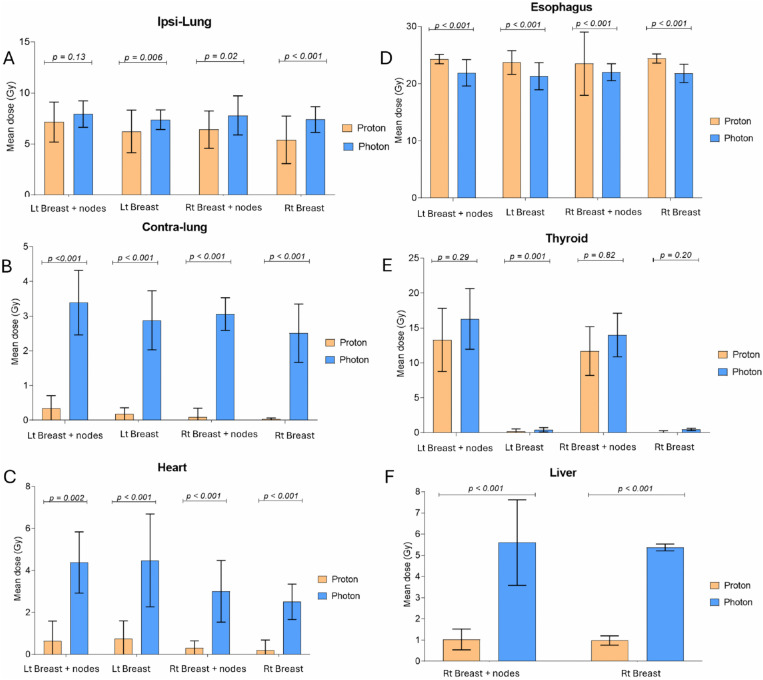
The mean dose to organs at risk with 95% confidence intervals, comparing proton (intensity modulated proton therapy, represented by blue bars) and photon (volumetric modulated arc therapy, represented by orange bars) for each subgroup: left breast with regional nodes, left breast only, right breast with regional nodes, and right breast only. A = ipsilateral lung, B = contralateral lung, C = heart, D = esophagus, E = thyroid, and F = liver (shown only for the right-sided subgroups).

Outcomes for the PTV and OARs dosimetric parameters are shown in Table 1. The representative dose distributions for IMPT and VMAT plans are illustrated in Fig 1. The mean dose of OARs in each subgroup are shown in Fig 2.

**Table 1:** The dosimetric comparison for the IMPT versus the VMAT plans.

Organs	Dose levels	Lt. breast + Regional nodes			Lt. breast			Rt. breast + regional nodes			Rt. breast		
		IMPT	VMAT	*P* value	IMPT	VMAT	*P* value	IMPT	VMAT	*P* value	IMPT	VMAT	*P* value
PTV	Dmax	28.9 ± 0.8	27.7 ± 0.9	<0.01	28.9 ± 1.1	27.6 ± 1.0	<0.01	28.2 ± 0.8	27.4 ± 0.51	<0.01	28.2 ± 0.9	26.6 ± 2.1	0.01
	D90	25.7 ± 0.2	25.5 ± 0.3	<0.01	25.7 ± 0.4	25.4 ± 0.3	<0.01	25.5 ± 0.4	25.4 ± 0.2	0.32	25.5 ± 0.4	25.3 ± 0.2	<0.01
	D95	24.8 ± 0.1	24.9 ± 0.3	0.85	24.8 ± 0.2	24.8 ± 0.2	0.85	24.7 ± 0.2	24.8 ± 0.3	0.43	24.8 ± 0.4	24.9 ± 0.6	0.59
Ipsilateral lung	Dmean	7.2 ± 2.0	7.9 ± 1.3	0.13	6.2 ± 2.1	7.4 ± 1.0	<0.01	6.4 ± 1.8	7.8 ± 1.9	0.02	5.4 ± 2.3	7.4 ± 1.3	<0.01
	V5 (%)	49.9 ± 11.7	54.9 ± 9.0	0.12	43.3 ± 11.9	50.1 ± 8.1	<0.01	43.7 ± 11.6	55.3 ± 9.7	<0.01	36.2 ± 14.3	48.9 ± 11.6	<0.01
	V10 (%)	32.0 ± 11.5	28.0 ± 6.3	0.18	26.8 ± 10.5	25.5 ± 4.2	0.51	28.9 (21.4-36.3)	28.7 (26.2 - 33.0)	0.68	24.9 (10.2-30.7)	24.4 (21.3-28.6)	0.09
	V20 (%)	6.1 (1.9-9.4)	8.2 (3.7-10.2)	0.48	3.8 (1.3-5.8)	6.1 (3.7-8.2)	0.29	6.5 (1.4-9.9)	6.6 (3.8-8.6)	0.68	5.4 (0.4-9.0)	5.5 (3.3-9.5)	0.44
Contra-lung	Dmean	0.4 (0.2-0.5)	3.4 (2.8-3.8)	<0.01	0.2 (0.1-0.3)	2.9 (2.4-3.2)	<0.01	0.1 (0.1-0.3)	3.1 (2.8-3.3)	<0.01	0 (0-0.1)	2.5 (2.0-2.8)	<0.01
	V5 (%)	1.1 (0.6-2.1)	17.0 (12.4-23.8)	<0.01	0.3 (0-0.9)	11.2 (7.0-17.3)	<0.01	0 (0-0.3)	14.9 (12.4 - 18.9)	<0.01	0 (0−0)	9.5 (6.4-11.2)	<0.01
	V10 (%)	0.3 (0.1-0.4)	1.0 (0.6-2)	<0.01	0 (0−0)	0.5 (0.1-1.2)	<0.01	0 (0−0)	0.3 (0-1)	<0.01	0 (0−0)	0.2 (0-0.4)	<0.01
Heart	Dmax	12.5 (9.1-17.6)	17.2 (13.7-20.2)	<0.01	13.6 (9.2-18.5)	18.3 (13.9-19.0)	0.02	6.3 (4.7-10.1)	9.5 (7.4–11.0)	0.07	4.7 (1.6-9.4)	10.1 (7.1-12.6)	<0.01
	Dmean	0.6 (0.5-1.4)	4.4 (3.2-4.7)	<0.01	0.8 (0.5-1.4)	4.5 (3.1-5.4)	<0.01	0.3 (0.2-0.5)	3.0 (2.3-3.8)	<0.01	0.2 (0.1-0.6)	3.2 (2.1-3.6)	<0.01
	V5 (%)	6.4 (3.3-10.5)	25.0 (14.8-27.7)	<0.01	5.2 (3.2-9.7)	24.6 (13.2-36.6)	<0.01	1.5 (0.6-2.7)	12.4 (5.9-21.4)	<0.01	0.7 (0-2.9)	16.3 (3.3-18.2)	<0.01
	V10 (%)	1.6 (0.8-4.2)	7.0 (4.0-9.3)	<0.01	2.2 (0.9-4.3)	7.7 (3.7-13)	<0.01	0.2 (0-1.2)	1.24 (0-2.0)	0.09	0 (0-0.4)	1.1 (0-1.8)	0.03
	V20 (%)	0.1 (0-0.5)	0.4 (0-0.7)	0.04	0 (0-0.5)	0.3 (0-0.6)	0.03	0 (0−0)	0 (0−0)	0.33	0 (0−0)	0 (0−0)	0.28
Skin	Dmax	28.4 ± 1.0	26.8 ± 0.9	<0.01	28.2 ± 1.4	26.6 ± 1.0	<0.01	28.0 ± 1.0	26.5 ± 0.6	<0.01	28.0 ± 1.1	26.3 ± 0.8	<0.01
	Dmean	24.3 ± 0.8	21.9 ± 2.3	<0.01	23.7 ± 2.1	21.4 ± 2.4	<0.01	23.5 ± 5.5	22.0 ± 1.5	0.21	24.4 ± 0.8	21.9 ± 1.6	<0.01
Esophagus	Dmax	23.0 ± 3.6	20.7 ± 6.1	0.02	0.9 (0.2-3.1)	8.3 (5.7-9.2)	<0.01	17.2 ± 5.7	17.6 ± 4.6	0.59	0.6 (0.1-2.6)	6.7 (5.3-9.3)	<0.01
	Dmean	4.0 ± 1.5	7.0 ± 2.8	<0.01	0.1 (0-0.3)	2.9 (2.3-3.4)	<0.01	2.51 ± 1.3	5.8 ± 1.0	<0.01	0.1 (0-0.2)	2.7 (2.2-3.1)	<0.01
Thyroid	Dmax	26.7 ± 1.7	26.0 ± 3.7	0.29	1.2 (0.6-2.8)	0.7 (0.5-1.0)	<0.01	24.3 ± 5.2	24.4 ± 4.8	0.82	0.4 (0.1-1.5)	0.7 (0.6-0.9)	0.2
	Dmean	13.4 ± 4.5	16.4 ± 4.3	0.10	0.2 (0.1-0.5)	0.4 (0.3-0.6)	0.04	11.7 ± 3.5	14.0 ± 3.1	<0.01	0.1 (0-0.2)	0.5 (0.4-0.5)	<0.01
Contralateral Breast	Dmax	1.18(0.77-2.55)	7.23(5.94-8.49)	<0.01	0.81(0.52-2.97)	6.39(4.71-8.06)	<0.01	1.68(1.29-2.27)	8.65(7.71-10.13)	<0.01	1.73(1.4-3.13)	8.55(6.88-9.84)	<0.01
	Dmean	0.07(0.06-0.023)	3.11(2.78-3.52)	<0.01	0.08(0.03-0.26)	2.89(2.64-3.26)	<0.01	0.10(0.09-0.20)	3.26(2.87-3.65)	<0.01	0.13(0.1-0.36)	3.43(2.57-3.55)	<0.01

The dose unit, GyRBE for IMPT and Gy for VMAT; Mean ± standard deviation for normally distributed data using a paired t-test; median (interquartile range) for non-normally distributed data using the Wilcoxon signed-rank test. Grey highlights indicate a significant difference (p-value < 0.01) between IMPT and VMAT within each subgroup.

Abbreviation: IMPT = intensity modulated proton therapy, VMAT = volume modulated arc therapy, PTV = planning target volume

### PTV dosimetry

PTV Dmax was significantly higher in IMPT among all scenarios and above the limit of 107%. PTV D90 was significantly lower in almost all VMAT plans, except for the right breast with regional nodes. PTV D95 was observed to be comparable for all plans.

### Ipsilateral lung dosimetry

The ipsilateral lung Dmean and V5 doses were significantly lower in IMPT methods for breast-only radiotherapy and for right breast with regional nodes. In all four scenarios, there was no significant difference between V10 and V20 for both IMPT and VMAT plans.

### Contralateral lung dosimetry

All IMPT plans demonstrated significantly reduced doses of Dmean, V5, and V10 to the contralateral lung dosimetry. IMPT Dmean was below 1 Gy on average. V5 was markedly higher in VMAT plans.

### Heart dosimetry

Dmean and V5 were significantly reduced with IMPT techniques in every scenario. Dmean was below 1 Gy for the left breast and below 0.5Gy for the right breast in IMPT plans. Dmax and V10 were significantly decreased in the IMPT for the left breast with or without regional nodes compared to the right breast. There was a significant difference in V20 between IMPT and VMAT in left breast only and left breast with regional nodes, but not between two plans of the right breast with and without regional nodes.

### Contralateral breast

Dmax and Dmean were minimized when using IMPT techniques. Both of these parameters were significantly lower when compared to using photons rather than protons in each instance. The Dmean of the contralateral breast consistently remained below 1 Gy in IMPT plans.

### Skin dosimetry

Skin dose was higher for IMPT plans. Dmax was significantly different for every scenario. Dmean was significantly different for all scenarios except for the right breast with regional nodes scenario.

### Esophagus dosimetry

The only plan in which there was no substantial difference in Dmax between IMPT and VMAT was right breast with regional node. The IMPT Dmax was substantially greater in the left breast with regional nodes plan. For breast-only scenarios, both Dmean and Dmax for IMPT were significantly reduced. Dmean was substantially lower in all IMPT cases.

### Thyroid dosimetry

There was no significant difference between two plans for left breast and regional nodes. Dmax and Dmean differed significantly for left breast. Dmax did not differ between IMPT and VMAT for the right breast with and without regional nodes, but Dmean was significantly lower in IMPT for right breast with and without reginal nodes. However, the absolute differences in dose were very small.

## Discussion

While dosimetric comparisons between proton and photon techniques have been reported in the context of conventional or moderately hypofractionated whole breast irradiation, data in the setting of ultrahypofractionated whole breast irradiation, particularly for cases including nodal irradiation, remain limited. Although this is a dosimetric study, it contributes to a better understanding and showes that IMPT offers superior sparing of several OARs compared to VMAT in the ultrahypofractionated setting. IMPT significantly reduced mean doses to the heart, lung, contralateral breast, thyroid, and esophagus, regardless of tumor laterality or nodal irradiation, except in left-side with nodal irradiation, where ipsilateral lung and thyroid mean doses did not differ significantly from VMAT. However, a notable trade-off was the higher skin dose observed with IMPT, with Dmax and Dmean significantly increased in nearly all plans.

Hypofractionation regimens in breast cancer have been widely adopted over the past decades [2,4,5], and moderate hypofractionation for both whole breast/chest wall and regional nodal radiotherapy has been shown to be safe and effective [6,7,17–20]. With the distinctive low α/β of breast cancer, high dose per fraction is suggested [21,22]. Therefore, ultra-hypofractionation regimen has been proposed and accepted as a treatment for whole breast radiotherapy in early-stage breast cancer [8,23]. Moreover, the clinical outcomes of ultrahypofractionation including regional nodes are awaited. A previously published study from our institution comparing post-mastectomy VMAT and IMPT in a hypofractionated setting demonstrated similar findings to those presented in this manuscript. However, due to the major differences in target volume, specifically, the thickness of the intact breast versus the chest wall, the DVH outcomes were not entirely the same. Although it might seem that anatomical differences or variations in skin, soft tissue, and target volume thickness may have minimal impact in photon therapy, even with advanced techniques such as VMAT, these factors become a major concern in proton beam therapy. Proton beams are highly sensitive to variations in the size, shape, and thickness of the target volume, as well as to the anatomical structures along the beam path. Therefore, we believe that the dosimetric results from this study may differ from those of the previous study. As anticipated, we observed differences in the DVH of normal structures located directly beneath the target volume. Specifically, the ipsilateral lung DVHs in our study tended to be lower, while the heart DVHs in left-sided cases were generally higher compared to those in the post-mastectomy setting. In addition to target volume thickness, the curved anatomy of the intact breast may contribute to differences in dose distribution, particularly at the skin and heart-lung interface, compared with the flatter anatomy of chest wall in the post-mastectomy setting. Moreover, whole breast irradiation often requires additional consideration of cosmetic outcomes and skin toxicity. Therefore, it is essential to evaluate dose distribution specifically in the setting of intact breast irradiation. While findings from post-mastectomy dosimetric study may offer some references, we believe that data from post breas-conserving surgery more accurately reflect the anatomical considerations relevant to whole breast irradiation. In real clinical practice, treatment planning techniques may need to be adapted according to individual clinical contexts.

As late toxicities are sensitive to high dose per fraction [24], ultrahypofractionation should be used cautiously. The FAST study reported no significant late toxicity at 10 years, while the FAST-Forward trial, with a similar dose and fractionation regimen to our study, has not yet reported late toxicity. Given the favorable overall survival in breast cancer, survivors may still experience late effects. Heart disease is one of the most concerning radiotherapy related toxicities in breast cancer, especially left sided breast or including internal mammary node in the target volume. Rates of major coronary events increased linearly with the mean heart dose (MHD) by 7.4% per 1 Gy [25]. A previous study also suggested that the degree of hypofractionation contributed to increased cardiac mortality [24]. Our study, which, in regional nodal radiotherapy scenarios, included IMNs, found that cardiac dose was much lower in IMPT than VMAT. These findings may suggest a potential for reduced late cardiotoxicity with proton therapy, particularly in cases where regional nodal irradiation is indicated. However, whether the dosimetric benefits can be translated into clinical outcomes remains unclear.

In present study, a subset of left-sided breast cancer cases employed deep inspiration breath hold (DIBH). DIBH is one of the techniques for reducing the risk of cardiac toxicity. We did not use DIBH in the proton scenario, as the objective was to observe the actual dose received by the heart, and the implementation of DIBH in our center was not considered for proton treatment. A retrospective study showed that the heart dose was lower in free-breathing proton therapy than in DIBH VMAT [25]. The findings of our study consistently demonstrated that IMPT had a greater reduction in cardiac dose compared to DIBH photon therapy. Moreover, increasing lung volumes from DIBH could worsen range uncertainty [26]. For these reasons, we did not employ DIBH in our study.

Hypothyroidism also occurred frequently in breast cancer patients who receive both conventional and hypofractionated radiotherapy [27,28]. The mean dose to the thyroid gland is used as a threshold to predict hypothyroidism, and our study showed that it was lower in proton therapy than in VMAT. However, to our knowledge, no clinical results have been reported yet for the setting of ultrahypofractionation. Our study showed that the Dmean of thyroid in IMPT was significantly lower than that in VMAT across almost all subgroups. Given the small difference observed, it remains uncertain whether this will translate into a clinical outcome.

Not only late toxicities, but acute toxicities also interrupt the treatment or affect patients’ quality of life. Our study showed that IMPT reduced dose to lung. This may suggest that the risk of radiation pneumonitis might decrease [29,30]. Risk of radiation pneumonitis occurred in less than 5% for the three-dimensional era of radiotherapy. However, this event needs proper treatment and investigation. Signs, symptoms, and X-ray images can mimic pulmonary infection or other inflammation. Bronchoscope is occasionally required which is an invasive procedure. Moreover, after resolving, lung fibrosis could develop. Although it may or may not affect pulmonary function, the fibrosis can also obscure lung lesions of X-ray images. In our study, V5 of ipsilateral lung was lower in IMPT planning although it was not significant in left breast with regional nodes scenario. However, it was significant for IMPT to reduce contralateral lung dose in all scenarios. These findings suggest that proton treatment may allow better sparing of normal lung tissue compared with VMAT, although clinical implications remain to be established. Our results are consistent with previous dosimetric study [31].

According to the recommendations from PTCOG, it is advised to restrict the average dose to all regions of the contralateral breast to less than 1 Gy, especially in women who are younger than 40 years old. [32] The Women’s Environmental, Cancer, and Radiation Epidemiology (WECARE) study found that found that women under 40 who received a mean breast dose above 1.0 Gy in specific regions had a 2.5-fold higher risk of developing contralateral breast cancer compared with unexposed women [33]. Minimizing radiation exposure to the contralateral breast is an important goal in treatment planning. In our study, IMPT plans delivered lower doses to the contralateral breast, likely due to beam arrangements and reduced scattered dose in proton therapy. Dosimetric modeling studies suggest this could reduce the lifetime risk of contralateral second primary breast cancer compared with photon-based IMRT [34–35]. However, clinical confirmation is needed.

An important finding of our study is that, despite lower Dmean with proton therapy, Dmax was generally higher than VMAT. In correspondence with our previous study, proton beam showed higher Dmax to skin compared with VMAT. Unlike passive scattering technique, the skin dose of IMPT can be optimized. Even though we subtracted target volumes by 5 mm from the skin surface, Dmax of skin in proton beam group was still high. One possible contributing factor may be the range shifter thickness. In our study, we used a 5-cm range shifter. The use of a 3-cm range shifter instead might help reduce the issue of high Dmax to the skin. Moreover, better optimization parameters selection, and the minimum distance between snout and patient surface, could be helpful. Further studies adapting these methods and parameters may help confirm our hypothesis. In addition, Dmax to the esophagus was higher with proton therapy in left breast with regional nodes. However, these effects typically resolve soon after treatment. For the results of FAST-FORWARD study, there is only mild skin reaction and no report of esophagitis in the 26 Gy per 5 fractions group, which we adopt as a regimen in our study [8]. Furthermore, recent data from clinical studies using ultrahypofractionated regimens consistently demonstrated that mostly only low-grade skin toxicities developed [36–38]. For proton based-ultrahypofractionation, a published abstract reported only low-grade skin toxicities [39]. However, a fully published manuscript is still lacking, particularly one that correlates with DVH outcomes. Theoretically, acute toxicities depend on total dose and treatment time rather than fraction size, so ultrahypofractionated regimens with lower total dose are expected to have favorable acute toxicity [40]. Despite relatively high skin Dmax percentages of our results, the absolute dose is low. Therefore, IMPT ultrahypofractionation–related skin toxicities are expected to be acceptable. Another preprinted randomized clinical study from our institution, comparing IMPT and VMAT in breast cancer patients receiving the same ultrahypofractionation regimen as prescribed in this manuscript, reported outcomes consistent with our findings. With short-term follow-up, the IMPT group experienced more dermatitis than the VMAT group. However, no high-grade dermatitis was observed [41].

Even though our study provided insights into proton and photon therapy in ultrahypofractionation after breast conserving surgery, there are some limitations. First, our sample size was too small to evaluate the difference among different breast sizes. In addition, all of our population is Asian, who mostly have smaller breast size than Western populations. The size of the target volume, Dmax of skin and dose of other parameters might be different. As previously mentioned, although dose distribution and doses to OARs in VMAT may not be substantially affected by breast size, proton therapy could be more directly influenced by these differences. Therefore, caution should be exercised when applying the results of this study to populations with larger breast sizes. Second, since this is the dosimetric study, the prospective clinical study with long-term follow-up is required to evaluate the clinical outcomes and the feasibility. Our findings revealed several significant differences in the DVH. In theory, we aim to achieve the lowest possible dose to normal organs. However, whether these differences will translate into clinical outcomes remains uncertain. Therefore, careful judgment is required when applying these findings in clinical practice. Third, the accessibility of proton therapy remains highly limited, and it is still considered a relatively new modality in many institutions. Therefore, the learning curve associated with proton therapy, which may lead to challenges in treatment planning, is difficult to avoid. As shown by our study, the relatively high skin Dmax could possibly be a consequence of our institution’s inexperience. Additionally, the cost of proton therapy is significantly higher due to factors such as infrastructure, equipment, and maintenance. Although we found that proton therapy can significantly reduce the dose to certain normal organs, the decision to use proton therapy for breast cancer must be carefully evaluated, taking into account the cost-effectiveness for the entire nation as well as the individual patient’s benefit.

In summary, our dosimetric analysis showed that IMPT reduced radiation dose to the heart, lungs and contralateral breast, while increasing skin dose compared to VMAT. These findings suggested a potential for reduced long-term toxicity with IMPT in the ultrahypofractionated setting. However, the clinical significance of these dosimetric differences remains to be established. Although skin toxicity may be higher with IMPT due to increased surface dose, it is generally short-term and manageable, but should still be carefully considered in clinical decision-making. As ultrahypofractionated radiotherapy becomes more widely adopted in breast cancer treatment, long-term follow-up data remains limited. Therefore, caution is warranted when applying these dosimetric findings to clinical practice. Dose to organs at risk should be minimized and thoroughly evaluated. Further prospective clinical studies are necessary to validate these dosimetric advantages and assess their impact on clinical outcomes.

## Supporting information

S1 Table The optimization goal used in the treatment planning. (DOCX)

## Abbreviations

CT: computed tomography
CTV: clinical target volume
DVHs: dose volume histograms
IMPT: intensity modulated proton therapy
PTV: planning target volume
OARs: organs at risk
VMAT: volumetric modulated arc therapy.
