# Supplementary material for: Dosimetric comparison study of ultrahypofractionated photon versus proton treatment plans in post breast-conserving surgery breast cancer
Source: PLoS One. 2026 Mar 11;21(3):e0344699. doi: 10.1371/journal.pone.0344699 (PMC12978489; doi:10.1371/journal.pone.0344699)
Supplement: S1 Table — (DOCX) [file pone.0344699.s001.docx]

**Supporting information Table 1:** The optimization goal used in the treatment planning

| Organ | Criteria | |
| --- | --- | --- |
| PTV | Dmax | < 28.6 Gy |
|  | D90% | ≥ 25.5 Gy |
|  | D95% | ≥ 24.7 Gy |
| Ipsi-Lung | Dmean | < 6 Gy |
|  | V5Gy | < 35% |
|  | V10Gy | < 25% |
|  | V20Gy | < 15% |
| Contra-Lung | Dmean | < 3 Gy |
|  | V5Gy | < 11% |
|  | V10Gy | < 0.5% |
| Heart | Dmax | < 5 Gy |
|  | Dmean | < 1 Gy |
| Surface | Dmax | < 26.5 Gy |
| Esophagus | Dmax | < 8 Gy |
| Breast | Dmax | <2 Gy |
|  | Dmean | < 0.5 |
